# Supplementary material for: A Critical View of the Application of the APEX Software (Aqueous Photochemistry of Environmentally-Occurring Xenobiotics) to Predict Photoreaction Kinetics in Surface Freshwaters
Source: Molecules. 2019 Dec 18;25(1):9. doi: 10.3390/molecules25010009 (PMC7017383; doi:10.3390/molecules25010009)
Supplement: Supplementary file 1 [file molecules-25-00009-s001.zip › 3.2.4_gcc-4.4.0.zip!/3.2.4_gcc-4.4.0/3.2.4_gcc-4.4.0/doc/octave/html/interpreter/Table-of-Output-Conversions.html]

Table of Output Conversions - Untitled


Next: Integer Conversions,
Previous: Output Conversion Syntax,
Up: C-Style I/O Functions


---

#### 14.2.7 Table of Output Conversions

Here is a table summarizing what all the different conversions do:

%d, %i: Print an integer as a signed decimal number. See Integer Conversions, for details. %d and %i are synonymous for output, but are different when used with `scanf` for input (see Table of Input Conversions). %o: Print an integer as an unsigned octal number. See Integer Conversions, for details. %u: Print an integer as an unsigned decimal number. See Integer Conversions, for details. %x, %X: Print an integer as an unsigned hexadecimal number. %x uses lower-case letters and %X uses upper-case. See Integer Conversions, for details. %f: Print a floating-point number in normal (fixed-point) notation. See Floating-Point Conversions, for details. %e, %E: Print a floating-point number in exponential notation. %e uses lower-case letters and %E uses upper-case. See Floating-Point Conversions, for details. %g, %G: Print a floating-point number in either normal (fixed-point) or exponential notation, whichever is more appropriate for its magnitude. %g uses lower-case letters and %G uses upper-case. See Floating-Point Conversions, for details. %c: Print a single character. See Other Output Conversions. %s: Print a string. See Other Output Conversions. %%: Print a literal % character. See Other Output Conversions.

If the syntax of a conversion specification is invalid, unpredictable
things will happen, so don't do this. If there aren't enough function
arguments provided to supply values for all the conversion
specifications in the template string, or if the arguments are not of
the correct types, the results are unpredictable. If you supply more
arguments than conversion specifications, the extra argument values are
simply ignored; this is sometimes useful.
